# Fully Convolutional Deep Neural Networks with Optimized Hyperparameters for Detection of Shockable and Non-Shockable Rhythms

**DOI:** 10.3390/s20102875

**Published:** 2020-05-19

**Authors:** Vessela Krasteva, Sarah Ménétré, Jean-Philippe Didon, Irena Jekova

**Affiliations:** 1 Institute of Biophysics and Biomedical Engineering, Bulgarian Academy of Sciences, Acad. G. Bonchev Str. Bl 105, 1113 Sofia, Bulgaria; vessika@biomed.bas.bg; 2 Schiller Médical, 4 Rue Louis Pasteur, 67160 Wissembourg, France; Sarah.MENETRE@schiller.fr (S.M.); Jean-Philippe.DIDON@schiller.fr (J.-P.D.)

**Keywords:** ECG, deep learning, convolutional neural networks, shock advisory system, life-threatening arrhythmias, ventricular fibrillation, out-of-hospital cardiac arrest

## Abstract

Deep neural networks (DNN) are state-of-the-art machine learning algorithms that can be learned to self-extract significant features of the electrocardiogram (ECG) and can generally provide high-output diagnostic accuracy if subjected to robust training and optimization on large datasets at high computational cost. So far, limited research and optimization of DNNs in shock advisory systems is found on large ECG arrhythmia databases from out-of-hospital cardiac arrests (OHCA). The objective of this study is to optimize the hyperparameters (HPs) of deep convolutional neural networks (CNN) for detection of shockable (Sh) and nonshockable (NSh) rhythms, and to validate the best HP settings for short and long analysis durations (2–10 s). Large numbers of (Sh + NSh) ECG samples were used for training (720 + 3170) and validation (739 + 5921) from Holters and defibrillators in OHCA. An end-to-end deep CNN architecture was implemented with one-lead raw ECG input layer (5 s, 125 Hz, 2.5 uV/LSB), configurable number of 5 to 23 hidden layers and output layer with diagnostic probability *p* ∈ [0: Sh,1: NSh]. The hidden layers contain N convolutional blocks × 3 layers (Conv1D (filters = Fi, kernel size = Ki), max-pooling (pool size = 2), dropout (rate = 0.3)), one global max-pooling and one dense layer. Random search optimization of HPs = {N, Fi, Ki}, i = 1, … N in a large grid of N = [1, 2, … 7], Fi = [5;50], Ki = [5;100] was performed. During training, the model with maximal balanced accuracy BAC = (Sensitivity + Specificity)/2 over 400 epochs was stored. The optimization principle is based on finding the common HPs space of a few top-ranked models and prediction of a robust HP setting by their median value. The optimal models for 1–7 CNN layers were trained with different learning rates LR = [10^−5^; 10^−2^] and the best model was finally validated on 2–10 s analysis durations. A number of 4216 random search models were trained. The optimal models with more than three convolutional layers did not exhibit substantial differences in performance BAC = (99.31–99.5%). Among them, the best model was found with {N = 5, Fi = {20, 15, 15, 10, 5}, Ki = {10, 10, 10, 10, 10}, 7521 trainable parameters} with maximal validation performance for 5-s analysis (BAC = 99.5%, Se = 99.6%, Sp = 99.4%) and tolerable drop in performance (<2% points) for very short 2-s analysis (BAC = 98.2%, Se = 97.6%, Sp = 98.7%). DNN application in future-generation shock advisory systems can improve the detection performance of Sh and NSh rhythms and can considerably shorten the analysis duration complying with resuscitation guidelines for minimal hands-off pauses.

## 1. Introduction

Cardiac arrest describes the loss of mechanical cardiac function and the absence of systemic circulation, presenting electrocardiographically (ECG) recorded rhythms of ventricular fibrillation (VF), rapid ventricular tachycardia (VT), asystole (ASYS), or pulseless electrical activity. Guidelines for resuscitation recommend prompt and effective bystander basic life support, uninterrupted, high-quality chest compressions, and early defibrillation for improved survival after cardiac arrest [1]. Only VT/VFs potentially receive benefit from defibrillation with post-shock return of spontaneous circulation, and they are the initial rhythms seen in 10% to 30% of the out-of-hospital cardiac arrests (OHCA) [2,3]. Non-VT/VF rhythms must not be shocked because no benefit will follow and deterioration in rhythm may result [4]. The reliable and prompt detection of cardiac rhythms as shockable (Sh) or nonshockable (NSh) is the primary requirement to the shock advisory algorithms in automatic external defibrillators (AEDs). Therefore, the AED performance goals on artifact-free ECGs are demanding (>90% for VFs, >75% for VTs, >95% for NSh, and >99% for normal sinus rhythms (NSR)), as set by the American Heart Association (AHA) in the early 1997 [4]. Besides, the hands-off pauses in chest compressions required for artifact-free ECG analysis in AEDs should be shortened, considering that 5 s to 10 s delay of the shock after stopping chest compressions reduces the probability of the defibrillation success and survival [5,6]. Therefore, effective strategies for early shock decision have been reported within the AED setting, such as early starting of the ECG analysis at the end of chest compressions [7] or during ventilation pauses [8]; as well as short ECG analysis durations, varying across studies from 2 s to 10 s [3,7,9,10,11,12,13,14,15,16,17,18,19,20,21,22,23,24,25,26,27].

During the last decades, Sh/NSh rhythm detection strategies employ comprehensive measurements of the ECG waveform morphology and heart rhythm periodicity in the time-domain [7,9,11,14,16,18,19,20,21,22,23,24,25,28,29], specific frequency bands via band-pass filtering for QRS or VF enhancement [11,13,14,15,21,22,23,30], Fourier transform [11,14,22,23,24,26,31,32] or time-frequency ECG transformations [10,24,27,33], as well as nonlinear ECG measures [11,12,14,17,22,23,24,34,35]. Although sets of those classical features measured with computer-based programs have been shown to present good discrimination between Sh/NSh rhythms with state-of-the-art machine learning classifiers (discriminant analysis, logistic regression, bagging and random forests, support vector machines, genetic algorithms) [15,21,22,23,24,26,27,35], the strict features measurement within the AED setting is a challenge. The AED limitations concern real-time analysis with minimal decision delay, low complexity and low memory requirement for computations that present a certain risk of poor feature quality due to simplified measurements, inaccurate delineation of ECG waves, filtering or approximations. Thus imprecise extraction of ECG features might limit AED performance despite powerful classification algorithms.

Recently, powerful tools have been developed in the field of deep learning, helping classification of biosignals by end-to-end architectures of deep neural networks (DNNs) [36,37]. Transferring this knowledge to ECG signals, DNN can accept raw ECG data as input and output diagnostic probabilities by self-extracting significant features that characterize different arrhythmia classes. In a deep hierarchical structure, the learned features tend to become more abstract as the network gets deeper [38]. Convolutional neural networks (CNNs), including hidden layers of convolutional filters with pretrained weights can be run in real-time, thus being feasible for different ECG monitoring applications, such as denoising [39,40], QRS detection [41,42], ECG segmentation [43], heartbeat classification [44,45,46,47,48], and arrhythmia classification with different output diagnosis labels (normal rhythm, atrial fibrillation, other rhythm, noise [49,50,51,52]; normal rhythm, atrial fibrillation, atrial flutter, ventricular fibrillation [53,54]). While the above studies use DNN architectures with 3 to 11 hidden layers, a recent study of Hannun et al. (2019) [55] has demonstrated that an end-to-end 34-layer DNN can classify a broad range of 12 distinct arrhythmias with high diagnostic performance similar to that of cardiologists.

In the focus of AED rhythm analysis, a few recent studies have been found to apply DNNs for detection of life-threatening arrhythmias. Related to the problem for detection of pulseless and pulsatile rhythm, Elola A et al. (2019) [56] show the superiority of two end-to-end DNN architectures (up to five CNN layers and one recurrent layer) vs. a classic machine learning approach (hand-crafted features and classifier) with analysis over 2–5 s on ECGs acquired by defibrillation pads from OHCA databases. The other problem for Sh/NSh rhythm detection has been addressed by four studies, proposing DNN architectures [57,58,59,60]. Nguyen and Kiseon (2018) [57] show the partial benefit of CNN, implementing three CNN layers instead of conventional feature extraction, but keeping the conservative approach with ECG preprocessing and support vector machines classifier. Acharya et al. (2018) [58] demonstrate that end-to-end CNN architecture (four CNN layers and three fully connected layers) is able to detect very short Sh/NSh segments (of only 2 s in duration). A limitation of the above two studies is the DNN training and reporting results on public Holter databases, while there is evidence that ECGs gathered by Holters and defibrillators during treatment in OHCA may be very different both for Sh and NSh rhythms [24,60]. OHCA databases are, however, proprietary, and we find only two DNN models trained on OHCA ECGs, i.e., Picon et al. (2019) [60] with two CNN layers and one long short-term memory (LSTM) layer; Irusta et al. (2019) [59] with two or three CNN layers and two dense layers. Both studies are shown to be compliant with AHA performance goals using short ECG segment lengths (2 s to 8 s). However, the object for DNN depth and hyperparameter optimization has not been in the scope of any study for shock advisory decision.

The objective of this study is to optimize the hyperparameters of an end-to-end fully convolutional DNN architecture (one to seven CNN layers, 5 to 23 hidden layers) for Sh/NSh rhythm detection using single-lead raw ECG signals from public Holter and OHCA databases with life-threatening arrhythmias. The validation performance of our best model is reported for short and long analysis durations (from 2 s to 10 s). Comparison to other published studies aims to prove the superiority of our DNN design. While the network can be run alone, without the need for preprocessing, waveform measurements, transformations or other machine learning algorithms, it has perspectives for certain applications in unsupervised database annotation and diagnosis platforms, as well as in reliable real-time AED shock-advisory systems in OHCA.

## 2. ECG Databases

The ECG data used in this study contains a wide variety of nonshockable and shockable rhythms extracted from two sources: public Holter ECG databases from continuously monitored patients with ventricular arrhythmias, and OHCA databases recorded by AEDs from patients in cardiac arrest.

### 2.1. Public Holter Databases

The full-length ECG recordings of three publicly available databases are considered:

- AHA fibrillation database (AHADB) [61], including 30 min ECG recordings from 10 patients (files A8001 to A8010); only the first out of the two available ECG channels is used;
- Massachusetts Institute of Technology – Beth Israel Hospital (MIT-BIH) malignant ventricular ectopy database (VFDB) [62,63,64], including 35 min ECG recordings from 22 patients (files 418 to 430; 602, 605, 607, 609, 610, 611, 612, 614, and 615); only the first out of the two available ECG channels is used;
- Creighton University (CU) ventricular tachyarrhythmia database (CUDB) [64,65,66], including 8 min ECG recordings from 35 patients (files cu01 to cu35); only one ECG channel is available and used.

All ECG signals were stored at sampling frequency *fs* = 250 Hz. No additional filtering was applied, although ECGs might be prefiltered during their hardware acquisition in Holters. Each ECG recording was split in nonoverlapping 10 s strips, which are annotated following the rhythm annotation scheme (see Section 2.3). Data augmentation by overlap of extracted strips in long-term Holter databases has not been applied because extra replication of identical rhythms from the same patient produces artificially big datasets without introducing statistically valuable information for extraction of new diagnostic features. Indeed the training process on such databases could be substantially slowed down with no effect on improving the machine learning accuracy. 

### 2.2. OHCA Databases

The OHCA databases were collected during 959 interventions with a commercial AED (Fred Easy, Schiller Médical, Wissembourg, France) in the region of Paris and outlying areas in two nonoverlapping periods:

- November 2010–December 2010 (OHCA1 from 226 patients);
- June 2011–September 2011 (OHCA2 from 733 patients).

One-channel ECG signals were acquired via the defibrillation pads in the anterolateral position, filtered in a bandwidth (1–30 Hz) by the AED input hardware circuits for baseline drift and high-frequency noise suppression, and sampled at *fs* = 250 Hz. Ten-second ECG strips during AED analysis were extracted and annotated following the rhythm annotation scheme (see Section 2.3). 

### 2.3. Rhythm Annotation

**Figure 1:**
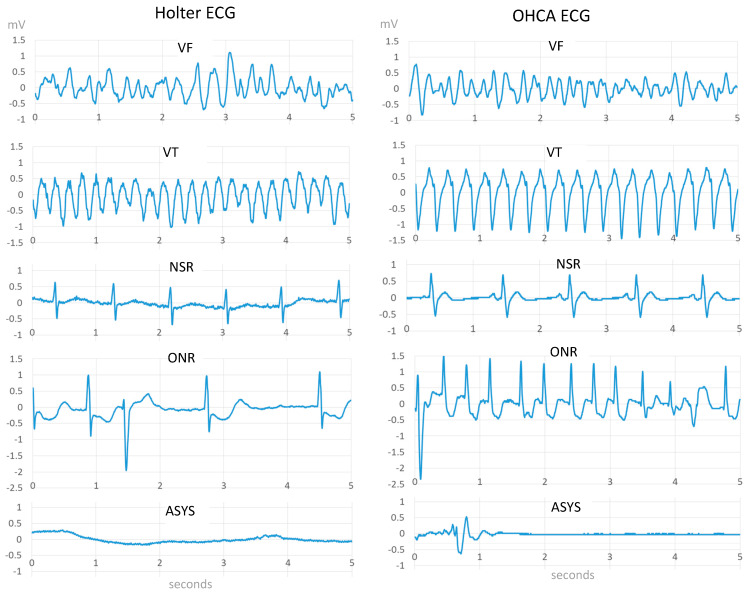
Examples of 5 s electrocardiogram (ECG) strips, extracted according to the defined annotation scheme for shockable (ventricular fibrillation—VF, rapid ventricular tachycardia—VT) and nonshockable (normal sinus rhythms—NSR, other nonshockable rhythms—ONR, asystole—ASYS) rhythms, found in Holter (left panel) and out-of-hospital cardiac arrests (OHCA) (right panel) databases.

The rhythm of each 10 s ECG strip was observed and independently annotated by three cardiologists. Majority voting was applied in cases of annotation disagreement. The annotations follow the AHA rhythm classification scheme [4], where performance goals are defined only in the absence of artifacts. Five basic Sh and NSh rhythm categories are defined (illustrated in Figure 1):

- Shockable rhythms, including:
  ○ Coarse ventricular fibrillation (VF) with amplitude >200 µV;
  ○ Rapid ventricular tachycardia (VT) with rate >150 bpm;
- Nonshockable rhythms, including:
  ○ Normal sinus rhythm (NSR) with visible P-QRS-T waves,
  ○ Other nonshockable rhythms (ONR), such as supraventricular tachycardia, sinus bradycardia, atrial fibrillation and flutter, heart block, idioventricular rhythms, and premature ventricular contractions;
  ○ Asystole (ASYS), representing ECG signal with peak to peak amplitude <100 µV, lasting more than 4 s;

This study excluded all 10 s strips that met one of the following conditions:

- Intermediate rhythms, consisting of fine ventricular fibrillations with amplitude in the range 100–200 µV (i.e., between ASYS and VF), and slow ventricular tachycardia with rate <150 bpm. AHA does not set any performance goal for such rhythms [4];
- Inconsistent rhythm (i.e., transition from NSh to Sh);
- Strips that contain extreme artifacts, significant baseline wander, electromyogram noise, pacemaker impulses.

### 2.4. Training/Validation Subsets

The annotated ECG strips were partitioned in two independent datasets, composed from different databases, as follows:

- A training dataset, including all Sh and NSh strips from AHA, CUDB and OHCA1 databases;
- A validation dataset, including all Sh and NSh strips from VFDB and OHCA2 databases.

The sample size of the training and validation datasets is presented in Table 1.

## 3. Methods

### 3.1. DNN Architecture

**Figure 2:**
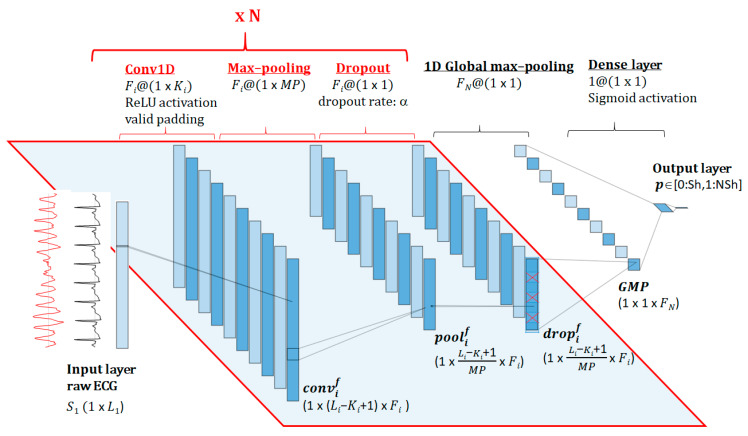
Еnd-to-end architecture of the proposed convolutional neural networks (CNN) model, showing input layer of raw ECG signal (one channel × length L_1_) followed by N consecutive blocks with a common fully-convolutional three-layer structure (1D convolution—Conv1D; max-pooling; dropout). The final diagnostic probability for Sh/NSh rhythm detection *p* ∈ [0: Sh, 1: NSh] is derived after global max pooling (GMP) and a dense layer binary classifier.

The architecture of the end-to-end fully CNN investigated in this study is presented in Figure 2. The input feature space is a 1D data vector read from one-lead raw ECG signal with size (1 × *L*_1_). It is next processed by a sequence of *N* convolutional blocks, each one including the three common layers of the CNNs architecture: 1D convolution (Conv1D), max-pooling and dropout [38]. The Conv1D layer of block number (*i)* consists of *Fi* filters with specific 1D convolution kernels of size (1 × *Ki*), thus providing *Fi* feature map representations of the ECG signal that maintain its temporal order [60]. The output of the *fth* filter (*f* = 1, 2,… *Fi*) is computed as:

(1) $$conv_{i}^{f}\left[j\right]=\left(\overset{Ki-1}{\underset{k=0}{\sum }}w_{ki}^{f}S_{i}\left[j+k\right]+b_{ki}^{f}\right)*A,$$

where:

- *i* = [1, 2, … *N*] identifies the sequential number of the convolutional layer.
- *S_i_* is the input vector of the *i^th^* convolutional layer, with size (1 × *Li*).
- *j* = [0, 1, … *Li* – *Ki* + 1] indexes the output feature vector, applying convolutional operation with a valid padding [37].
- $w_{ki}$ are the weights and $b_{ki}$ are the biases of the convolution kernel;
- *A* is the applied nonlinear activation function ReLU (rectified linear unit);

Next, the Conv1D layer output ($conv_{i}^{f}$) with size (1 × (*Li* − *Ki* + 1) × *Fi*) is downsampled by a max-pooling layer (pool size = (1 × *MP*)). It applies maximum operation over nonoverlapping segments of the feature vector $conv_{i}^{f}$, thus generating a new feature vector $pool_{i}^{f}$ with *MP* times smaller width (1 × $\frac{\mathrm{Li}-\mathrm{Ki}+1}{\mathrm{MP}}$ x Fi). To avoid overfitting and improve the generalization, a dropout regularization layer is applied during training with a dropout rate α ∈ [0; 1], thus generating an output vector $drop_{i}^{f}$ (1 × $\frac{\mathrm{Li}-\mathrm{Ki}+1}{\mathrm{MP}}$ × Fi) with portion of ‘0′ nodes equal to α. In the test process $drop_{i}^{f}=pool_{i}^{f}$. The input signal for the next convolutional layer is *S_i+1_* = $drop_{i}^{f}$. 

The sequence of N convolutional blocks with the structure described above is followed by a global max-pooling (GMP) layer, which downsamples $drop_{N}^{f}$ of each filter (*f* = 1, 2, … *F_N_*) to a single value equal to its maximal value. Thus the GMP layer has an output feature size (1 × 1 × *F_N_*), which is fed into a binary classifier implemented as a dense layer with a sigmoid activation function. The output layer provides the diagnostic probability for Sh/NSh rhythm detection *p* ∈ [0: Sh,1: NSh].

The trainable parameters in the proposed DNN architecture correspond to the weights and biases of the *N*-blocks Conv1D layers and the final dense layer, therefore, this model can be considered as a fully CNN. The number of trainable parameters can be calculated with the following equation:

(2) $$Params=\overset{N}{\underset{i=1}{\sum }}F_{i}{(K}_{i}F_{i-1}+1)+{(F}_{N}+1).$$

### 3.2. Hyperparameters Optimization

Although the structure of the designed DNN architecture implements a sequence of standard CNN layers (Figure 2), their basic hyperparameter (HP) settings are a priori unknown. These include:

- Number of sequential CNN blocks (*N)*, which virtually represents the depth of the network;
- Number of filters (*Fi*) and kernel size (*Ki*) of Conv1D in each sequential block (*i* = 1, 2, … *N*) that majorly influence the feature map representations of the ECG signal;
- Max-pooling size: A minimal fixed setting MP = 2 is used to gradually subsample the feature space at each sequential CNN block N, thus providing conditions to build deeper networks;
- Dropout rate: α = 0.3 is adopted as the most common dropout setting [37], based also on reports that values of α > 0.3 rapidly increase the error rate [56].

**Figure 3:**
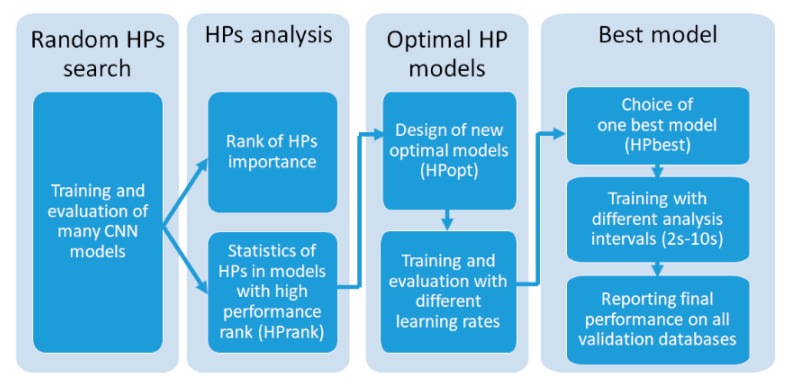
Process of hyperparameter (HP) search, analysis and optimization for justification of the best deep neural network (DNN) model.

We hypothesize that $HPs={\left\{N,F_{i},K_{i}\right\}}_{i=1}^{N},$ determining the number of trainable parameters (Equation (2)) might have significant influence on the Sh/NSh rhythm detection performance. Therefore, we performed a process of search, analysis and optimization of the top-ranked HP settings with the final aim to justify the choice of the best CNN model. The whole process is schematically summarized in Figure 3 and will be further described in detail. 

#### 3.2.1. Random HP Search

The first task of this study was to train different CNN models with settings of $HPs={\left\{N,F_{i},K_{i}\right\}}_{i=1}^{N},\;$ which were selected by random search in a large grid of HP values:

- *N* = {1, 2, 3, 4, 5, 6, 7};
- *Fi* = {5, 10, 15, 20, 25, 30, 40, 50}; additional range *F_1_* = {75, 100, 125, 150, 200} is included in the search space only for the shallowest CNN (*N* = 1), aiming to increase the number of trainable parameters to levels comparable to deeper CNNs (*N* > 1);
- *Ki* = {5, 10, 15, 20, 25, 30, 40, 50, 60, 70, 85, 100}; owing to the same reason as above, extra-large kernel sizes *K_1_*= {125, 150, 200} are included in the search space of CNN (*N* = 1);
- The vectors {*Fi*} and *{Ki*} are designed to follow a decreasing, increasing or constant trend from top to bottom layers (*i* = 1, 2, … *N)* in the same model.

The random HPs search was performed under equal training conditions for all models (defined in Section 3.3). The input ECG vector length was L_1_ = 5 s (625 samples@125 Hz), taken from the initial part (0–5 s) of the annotated ECG strips (10 s) in databases after downsampling by 2. The concept for selection of the initial signal part (without shifting) simulates the real-case scenario when the AED analysis process was started without a delay to output the earliest Sh/NSh decision. 

All random search CNN models are evaluated according to their performance on the validation dataset. The performance was estimated at the point on the receiver operating characteristic curve (ROC) with balanced sensitivity (Se) and specificity (Sp), i.e., the ROC point corresponding to maximal balanced accuracy (BAC):

(3) $$BAC=\frac{Se+Sp}{2}\to max$$

(4) $$Se=\frac{TP}{TP+FN},\;\;Sp=\frac{TN}{TN+FP}\;,$$

where *TP* are the correctly detected Sh cases; *FN* are the Sh cases classified as NSh; *TN* are the correctly detected NSh cases; *FP* are the NSh cases classified as Sh.

#### 3.2.2. HPs Analysis

Our first focus was to estimate the relative importance of each HP to the performance of all random search models. For this purpose, we applied a regression tree (RT) to predict the dependent variable (BAC) with continuous predictors $HPs={\left\{Params,F_{i},K_{i}\right\}}_{i=1}^{N},$ using the depth of the network as a covariate *N* = {1, 2, 3, 4, 5, 6, 7}. RT has the option to weight the predictors’ importance by calculating their importance score IS = [0; 1] based on the relative importance of the predictor in the full set of splits in the tree. Our RT design uses algorithms built into Statistica 12 (Dell Inc., Round Rock, Texas, USA). Computational details regarding this measure can be found in [67]. Note that the embedded concept of predictor importance is related to the method of surrogate splitting, which has the advantage of identifying variables that may contain important predictive power with respect to the outcome of interest, although they might be never chosen for any split due to colinearity with superior variables (e.g., we can achieve importance scores for all HPs, although *Params* are correlated to both *Ki* and *Fi*).

In the second step, we analyzed the statistical distributions of HPs (median value, quartile-range) using BAC performance of all random search models as a covariate. Then we focused on the small proportion of models with top-ranked performance *(P_R_)* with HPs denoted as:

(5) $$HPrank={\{HPs}^{P_{R}}\}\to \max\left(BAC\right),$$

which was our strategy for statistically justified HP optimization, as further communicated.

#### 3.2.3. Optimal HP Models

For different CNN depths *N* = {1, 2, 3, 4, 5, 6, 7}, the HPrank distributions (median values) were used to design new statistically justified “median HP” models which were expected to perform equally well to the top-ranked models. We further denoted them as our optimal CNN configurations, valid for different *N*:

(6) $$HPopt=median{(HPrank\left\{F_{i},K_{i}\right\}}_{i=1}^{N}).$$

All HPopt models were further trained under equal training conditions (defined in Section 3.3) and optimized for different learning rates *LR* = {0.01, 0.005, 0.001, 0.0005, 0.0001, 0.00005, 0.00001}, each *LR* used in 10 independent runs for model training. All trained HPopt models were further evaluated on the validation dataset. 

#### 3.2.4. Best Model

The settings of the best-performing HPopt model are reported as our best CNN configuration:

(7) $$HPbest=HPopt{\left\{LR,\;N,\;F_{i},K_{i}\right\}}_{i=1}^{N}\to \max\left(BAC\right).$$

CNN models with HPbest settings were further trained for different ECG analysis durations *L*_1_ = (2 s, 3 s, 4 s, 5 s, 7 s, 10 s), each one used in 10 independent runs for training. The evaluation of all trained models on the validation dataset justified our best CNN models for short and long analysis durations (2–10 s).

### 3.3. Training of DNN Models

All DNN models were programmed in the TensorFlow framework using Keras built-in APIs for model design, training, and evaluation. After the training was completed, the parameters of all networks were stored in HDF5 files. All experiments were conducted on a workstation with Intel(R)Xeon(R) CPU E5-2630 0 @ 2.30 GHz (2 processors), 32G B RAM, NVIDIA Quadro K4000-3Gb GPU. 

The following concepts were applied for management of the input data for model fitting:

- Keep balanced training dataset by replicating the shockable cases four times, considering the ratio of total NSh/Sh cases in Table 1, i.e., after replication the number of Sh cases (4 × 720 = 2880) becomes roughly equal to the number of NSh cases (3170);
- Shuffle the training data for randomization before feeding it into batches;
- Split the training data into batches, because using small batch sizes achieves the best training stability and generalization performance;
- Normalization of the input data is not applied and the input signal resolution of 2.5 μV/LSB is maintained. We purposely keep the real ECG amplitude, since it is characteristic for some of the analyzed rhythms (e.g., ASYS peak-to-peak amplitude <100 µV).

The following settings were applied for the model design and fitting:

- Training epochs: 400. Early stopping is applied if no improvement in performance is observed for more than 150 epochs;
- Batch size: 256;
- Kernel initializer: random uniform;
- Optimizer: ‘Adam’ with learning rate *LR* = 0.001, chosen as a good default setting [37], decay rate *DR* = *LR*/epochs, exponential decay rate for the first moment estimates β_1_ = 0.9 and exponential decay rate for the second moment estimates β_2_ = 0.999;
- Loss function: binary cross-entropy for 2 target classes (Sh/NSh);
- Metrics function: accuracy = (TP + TN)/(TP + TN + FP + FN). Owing to the concept for balanced training dataset during model fit, the metrics accuracy closely corresponds to BAC.
- Saved model: the model with maximal accuracy after all training epochs.

## 4. Results

### 4.1. Random HPs Search

The random search training of CNN models was automatically performed within the defined HP grid of values $HPs={\left\{N,F_{i},K_{i}\right\}}_{i=1}^{N}$ (Section 3.2.1), using a fixed setting of the network depth ${\left\{N=const\right\}}_{N=1}^{7}$ in one training session. A relatively similar duration of the training sessions has been respected, however, owing to some specifics in model training (mentioned below), the number of trained CNN models with distinct depths was globally different:

- *(N* = 1): 195 models, which cover the full search grid (13 filters × 15 kernel sizes). They are trained for 202 (120–298) epochs, reported as median value (quartile range). All models converged within 400 epochs.
- *(N* = 2): 1305 models trained for 140 (74–226) epochs. The relatively smaller number of trainable parameters than deeper networks resulted in a larger number of trained models generated during the training session;
- *(N* = 3): 707 models trained for 116 (57–204) epochs;
- *(N* = 4): 715 models trained for 69 (35–171) epochs;
- *(N* = 5): 716 models trained for 55 (25–143) epochs;
- *(N* = 6): 275 models trained for 44 (19–88) epochs;
- *(N* = 7): 303 models trained for 51 (20–62) epochs. Note the about 2.5-times smaller number of very deep models (*N* = 6, 7) than (*N* = 3, 4, 5), which is a consequence of the limited optional values for setting *K_i_* in deeper CNN layers owing to the effect of reaching maximal model shrink with valid padding. In some iterations, the random search algorithm has spent abundant amount of time for finding a valid HPs setting.

**Figure 4:**
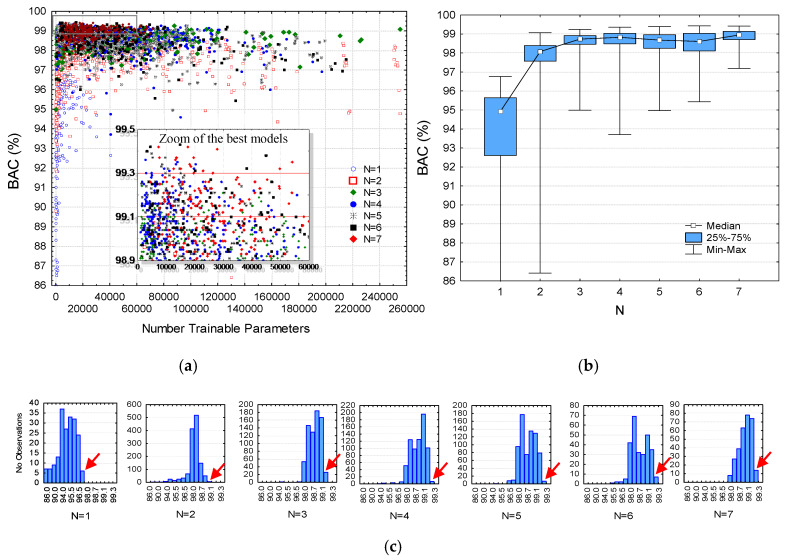
Analysis of validation balanced accuracy (BAC) performance for all CNN models trained with random search: (**a**) scatterplot of BAC in function of the number of trainable parameters; (**b**) box plots of BAC categorized to the network depth *N* = {1, 2, 3, 4, 5, 6, 7}; (**c**) BAC histograms categorized to N and highlighting the selected models with top-ranked performance (red arrow).

We illustrate the performance of all 4216 models generated by random HPs search in Figure 4—first shown as individual hits in BAC scatterplot (Figure 4a) and second as a statistical interpretation of those BAC hits in respect to the network depth *N* (Figure 4b). The dense overlapping scatterplots are seen as very similar BAC quartiles (fixed roughly in a 1% span within 98%–99%) and BAC min-max ranges (fixed roughly in a 5.5% span from about 95% to 99.5%) for all models with *N* ≥ 3. The shallowest models have a downshift performance, estimated within a BAC quartile range (97.6%–98.4%, *N* = 2) and (92.7%–95.7%, *N* = 1). Following the strategy for statistically justified HPs optimization, our focus was only on the top ranked performance models, which are zoomed in Figure 4a and pointed out (arrows) in the rightmost part of the BAC histograms in Figure 4c. According to the observations, we define four BAC thresholds for selection of the top-ranked models:

- BAC ≥ 96.5% applied for *N* = 1 (selecting six models);
- BAC ≥ 98.9% for *N* = 2 (five models);
- BAC ≥ 99.1% for *N* = 3 (26 models);
- BAC ≥ 99.3% for *N* = 4 (seven models), *N* = 5 (seven models), *N* = 6 (seven models), *N* = 7 (14 models).

### 4.2. HP Statistical Analysis

**Figure 5:**
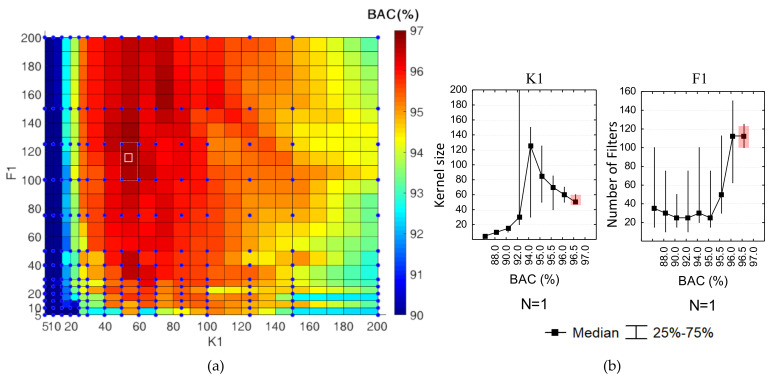
Analysis of $HPs=\left\{K_{1},F_{1},\right\}$ of random search CNNs with one convolutional block (*N* = 1):(**a**) Colormap of validation performance: $BAC=f\left\{K_{1},F_{1},\right\}$ generated in a fine surface grid by four-nearest-neighbors interpolation between the measurement points of the search grid (blue dots). The highlighted white zone covers the HPs of the top-ranked performance models, i.e., *HPrank* quartile range (rectangle) and *HPopt = HPrank* median value (square); (**b**) statistical distributions of $\left\{K_{1},F_{1},\right\}=f\left(BAC\right)$, presented as median values (dots) and quartile ranges (whiskers). *HPrank* quartile range of the top ranked performance models is highlighted in the rightmost distributions, corresponding to BAC ≥ 96.5%.

A simple example, which can visually justify the principle of our statistics-based HP optimization is illustrated in Figure 5a. It represents a 2D colormap $BAC=f\left\{K_{1},F_{1},\right\}$ for all models with one CNN layer (*N* = 1), which are trained for the full search grid in the 2D space $\;HPs\;\left\{K_{1},F_{1}\right\}\in \left[5;200\right]$. The colormap gradient clearly identifies that BAC is nonlinearly dependent on $HPs\;\left\{K_{1},F_{1}\right\},$ with a maximal performance red area in a relatively narrow mid part of $K_{1}=\left[30;85\right]$ and a wider range of high $F_{1}\;40$. However, this optimal area has no clear peak or contours because it is representative for the performance variance that each model can have in different training attempts. As soon as all models in the random search are trained only once, the performance of all models in the grid is randomly distributed within this variance. Therefore, all $HPs\;\left\{K_{1},F_{1}\right\}$ were adjusted to BAC, as shown in Figure 5b, where the common values in 50% of observations (quartile range and median value) were used to derive conclusions about the HPs distributions, which are associated with specific BAC range. These statistical distributions show clear nonlinear trends for $K_{1}=f\left(BAC\right)$ and $F_{1}=f\left(BAC\right)$, which have important implications on the analysis of the feature importance score, as further communicated. Our current focus is on the 50% of the top ranked performance models, which manifest very similar HPrank values, allocated in narrow quartiles—the red highlighted range in Figure 5b, translated to the white rectangle in Figure 5a, i.e., $HPrank{\{K}_{1}=\left[50;65\right],\;F_{1}=\left[100;125\right]\}.$ Our optimization criterion based on median(HPrank) (Equation (6)) predicts a new statistically justified model: $HPopt\{K_{1}=50,\;F_{1}=113\}$, depicted with the white square in Figure 5a. We observe that its coordinates fall in an interpolated red zone of high performance (between four points of our search grid with BAC = {96.50, 96.52, 96.65, 96.74}) and it is a subject of our further optimization results to prove that the real HPopt performance corresponds equally well to the predicted in this graph performance.

**Figure 6:**
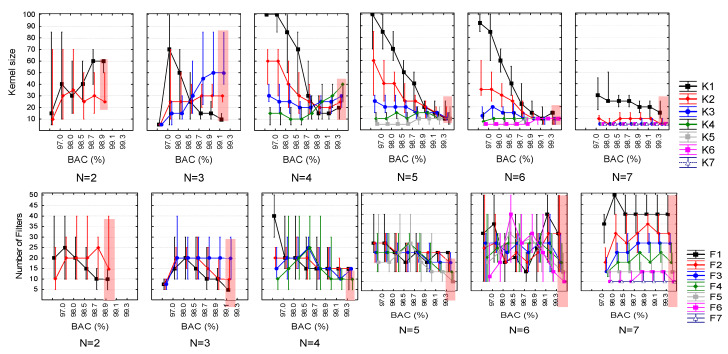
Distributions of $HPs={\left\{K_{i},F_{i},\right\}}_{i=1}^{N}$ of random search CNNs with more than one convolutional block (*N* = 2, 3… 7), corresponding to $K_{i}=f\left(BAC\right)$ (top plots) and $F_{i}=f\left(BAC\right)$ (bottom plots) as median values (dots) and quartile ranges (whiskers). *HPrank* quartile ranges of the top-ranked performance models are highlighted in the rightmost distributions.

**Figure 7:**
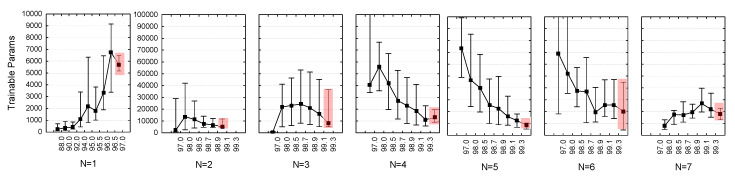
Number of trainable parameters in function of the validation BAC performance. The distributions are presented as median values (dots) and quartile ranges (whiskers). The number of parameters of the top-ranked performance models is highlighted in the rightmost distributions.

The above statistically justified principles were applied for the performance analysis in the multivariate search grid $HPs={\left\{K_{i},F_{i},\right\}}_{i=1}^{N}$ of deeper CNNs (N = 2, 3… 7) so that HP median values and quartile ranges were drawn in function of BAC (Figure 6). Our focus on the top-ranked models identified the quartile ranges of HPrank (highlighted red zone), and most importantly the median points, which were used to derive our optimal CNN configurations: HPopt=median(HPrank). We further considered the number of trainable parameters since they might have an important influence on the model training process and accuracy. They were subjected to the above statistical approach, adjusting their distributions in function of BAC (Figure 7). The graphs were used only for observational purposes and were not part of the model optimization settings HPopt=median(HPrank) as soon as *Params* were a derivation of the other HPs in the random search (Equation (2)): $Params=f{\left\{N,F_{i},K_{i}\right\}}_{i=1}^{N}$. The observations show that the top-ranked models (highlighted red zone in Figure 7) have limited trainable parameters, with quartile ranges of about 5000 to 45,000 parameters.

### 4.3. Rank of HPs Importance

Another result is the computed importance score of $HPs={\left\{Params,F_{i},K_{i}\right\}}_{i=1}^{N}$ as individual predictors of CNN performance, presented in Table 2. This importance score can be used for identifying the significant nonlinear trends of HPs=f(BAC), depicted in Figure 5b and Figure 6, Figure 7. The red highlighted cells in Table 2 (IS > 0.5) clearly indicate that the kernel sizes (*N*= 1, … 7) and the number of filters (*N* = 7) in the first convolutional layers, and consequently the number of CNN parameters (*N* = 1, …, 7) have significant importance to the BAC.

### 4.4. Optimal HP Models

**Figure 8:**
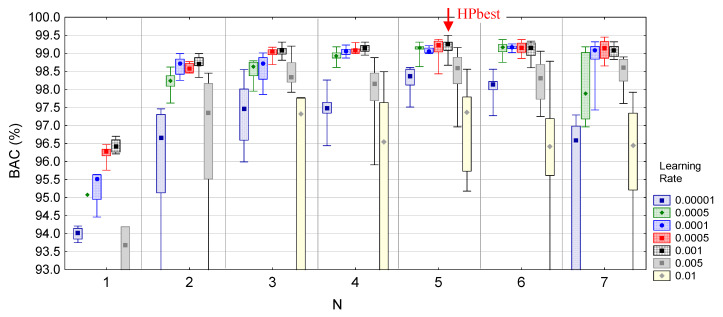
Validation BAC performance of *HPopt* models with different depths (*N* = 1, 2, … 7), trained with different learning rates. The distributions are presented as median values (dots), quartile ranges (boxes) and min–max range (whiskers). The red arrow highlights the best model, i.e., *N* = 5 (*LR* = 0.001), having BAC→max.

Table 3 presents the predicted optimal model settings $HPopt=median(HPrank{\left\{N,\;F_{i},K_{i}\right\}}_{i=1,\;}^{N}$, *N* = 1, … 7, which were derived from the highlighted top-rank ranges in Figure 5b and Figure 6. The performance of these optimal models is presented in Figure 8 for different learning rates. The min–max and quartile range distributions in Figure 8 show the natural BAC variance, which was observed between different training runs of a model with the same HPopt settings. We note that BAC variance is dependent on the *LR* and is minimal for *LR* = 0.0001–0.001 (<0.5%). Besides, we found that *LR* = 0.0001–0.001 is the optimal setting for training of the top-ranked HPopt models (BAC > 99% for *N* ≥ 3). The best performances of all HPopt models are reported in Table 3. Among them, our best model (BAC = 99.5) was found to be:

(8) HPbest={N=5, LR=0.001, F={20,15,15,10,5},K={10,10,10,10,10}}→max(BAC)

**Figure 9:**
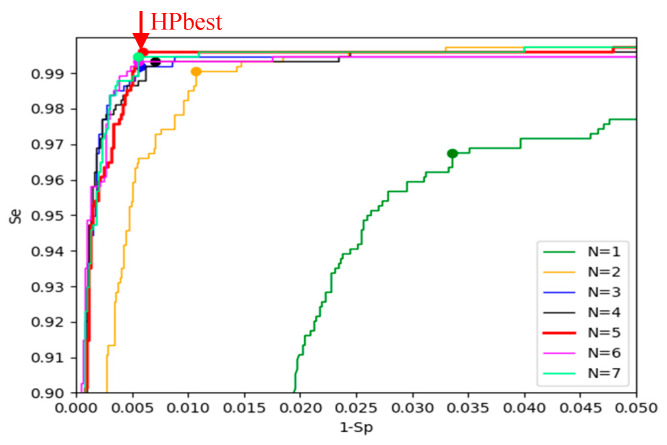
Validation receiver operating characteristic curves (ROC) of *HPopt* models with different depths (N = 1, 2 … 7). The dot marks correspond to the ROC point with maximal BAC (Se + Sp→max). The red ROC (*N* = 5) corresponds to the selected *HPbest*.

Figure 9 illustrates the validation ROC for all HPopt models in Table 3. We note that ROCs and their BAC points are closely overlapping for *N* ≥ 3 CNN layers, where BAC coordinates correspond to balanced Se and Sp in the same range (99%–99.6%). The red model with maximal BAC (HPbest) is distinguished to reach the highest Se.

### 4.5. Best Model

**Figure 10:**
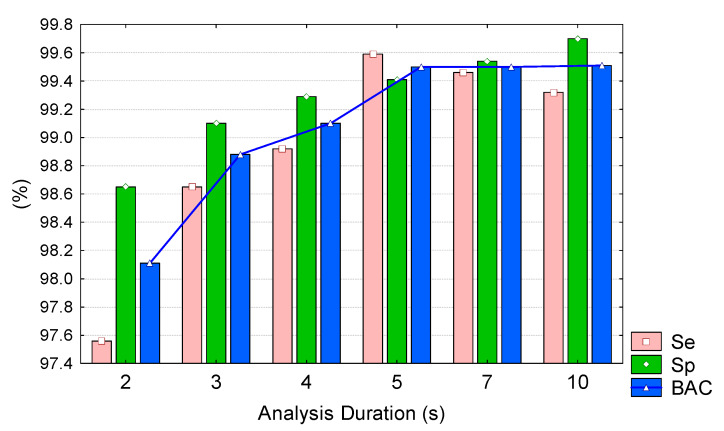
Validation sensitivity (Se), specificity (Sp), and BAC of our best CNN model (*HPbest*) for different analysis durations of the input ECG signal.

Figure 10 and Table 4 present the validation performance of our best model (HPbest) for different analysis durations (2–10 s) measured within the following ranges: Se (97.6%–99.6%), Sp (98.7%–99.7%), BAC (98.2%–99.5%). Maximal performance is measured at 5 s (maxSe = 99.6%) and 10s (maxSp = 99.7%). We note that BAC linearly increases from 25 s but further prolongation of the analysis duration (5–10 s) did not improve BAC owing to the effect of proportional Sp rise (+0.3% points) and Se drop (−0.3% points).

Table 4 also presents the final report of the validation performance on the Public and OHCA databases. Overall, the performance is lower in OHCA vs. Public databases, estimated with an average drop of −2.2% points for Se (95.2%–98.7% vs. 98.6%–100%), −0.3% points for Sp (98.7%–99.2% vs. 98.6%–99.8%), −1.2% points for BAC (97%–99% vs. 99.5%–99.9%).

## 5. Discussion

### 5.1. HPs Optimization

Although the detection of life-threatening cardiac arrhythmias has been handled over two decades with hand-crafted features and classic machine learning approaches, it is still a research area for improvements with special attention on maximal shortening of the analysis interval, thus providing the earliest shock advisory decision. This study addresses a substantially new approach for self-extracting significant features that characterize the raw ECG signals of different arrhythmias by means of end-to-end fully convolutional DNNs. The DNN input is fully consistent with the limited input setting for AED operation, without the need of additional pre-processing stages (e.g., filtering, feature extraction, signal transformations, gathering information from other ECG leads, etc.). This study contributes with results from the performed large scale HPs optimization $HPs={\left\{N,F_{i},K_{i}\right\}}_{i=1}^{N}$ by random grid search in a configurable DNN implementation (Figure 2), including:

- Shallow and very deep DNNs (from five to 23 hidden layers), composed by three to 21 layers from one to seven CNN blocks × three layers (Conv1D, max-pooling, dropout) + one GMP + one dense layer for binary classification;
- Different number of filters in each Conv1D layer: $F_{i}\in \left[5;50\right]$, *i* =1… *N*;
- Different kernel sizes in each Conv1D layer: $K_{i}\in \left[5;100\right]$, *i* = 1 … *N*, valid for *fs* = 125 Hz.

To the best of our knowledge, such large grid ranges of ${\{N,\;K}_{i},\;F_{i}\}$ are beyond the HP settings used in other published CNN studies for ECG diagnostic classification [56,57,58,59,60]. We consider that the performed HPs optimization process is computationally exhaustive and it has probably not been applicable or goes beyond the objective of other related works. The applied random search optimization is probably more computationally expensive and training time consuming than recently published DNN optimization algorithms based on neural architecture search (ENAS [68] and DARTS [69]), originally shown for image processing. In this study, we rely on the comprehensive interpretation of results from the classical random search, questioning whether the novel techniques, designed for search of optimal subgraphs with fixed blocks can deal with optimization of the hyperparameters in the blocks. This might be an interesting object of future research, transferring knowledge from image to ECG classification.

Our optimization goal is based on maximal BAC, which corresponds to the most convex point of ROC (Se + Sp→max) (Figure 9). This optimization score is beneficial for maximizing together both Se and Sp, as only those statistical indices have threshold requirements in the AHA performance goals [4]. Generally, BAC assumes equal proportion of false detections within both classes (Sh and NSh), leading to larger absolute number of FPs in the larger NSh class (5921 cases), which is about eight times larger than the Sh class (739 cases), i.e., BAC assumes imbalanced number of FP and FN, proportional to the class size (FP_BAC_ ≅ 8FN_BAC_). In contrast, other statistical metrics known to deal with imbalanced classes, such as F1-score = 2TP/(2TP + FP + FN) would assume balanced number of FP and FN (FP_F1_ ≅ FN_F1_) at the expense of increasing the number of FN_F1_>>FN_BAC_. Thus, using FN_F1_ in the denominator of Se could lead to an intolerable Se drop below the AHA goals [4].

Our results are distinctive to the general concept for optimal HPs design and the choice of the best performance model based on statistical analysis of a total of 4216 random search models. All models have been trained and evaluated under the same conditions with independent public and OHCA datasets for Sh/NSh classification over 5s ECG signals. Our initial observations reveal a large number of models, which have overlapping performances within the range (98%–99.5%), seen in the dense top-left corner of the BAC scatterplot (Figure 4a), as well as in the BAC boxplots and histograms (Figure 4b,c) of all deep networks with more than three CNN layers (*N* ≥ 3). The simplest optimization approach might take the best model from the random search and to just report its HPs settings and performance. We go further and define more generalized rules for optimal model predictions, answering to the following questions:

*(1) What is the common HP space between several top-ranked models?* The answer is highlighted in the quartile range distributions of $HPs{\left\{F_{i},K_{i}\right\}}_{i=1}^{N}=f\left(BAC\to max\right)$ Figure 5b and Figure 6. Our optimal design is then fitted to the median of those distributions, assuming a more robust HP setting derived from several models rather than just taking a single model. The proposed new “median HP” models are designed for *N =* 1, … 7 (Table 3), and they are proven to perform equally well to the top-ranked models in the random search (Figure 4).

*(2) Which are the HPs with major importance to the global CNN performance?* The answer to this basic question could considerably simplify the optimization problem in the multivariate HPs space of deep networks, giving the focus only to a few key HPs, which can potentially majorly influence the final outcome. Our interpretation is highlighted in Table 2, ranking the relative HPs importance with a regression tree for BAC prediction over statistics on all random search models: $IS{\left\{Params,F_{i},K_{i}\right\}}_{i=1}^{N}\;\in \left[0;1\right]$. In general, the most important features to BAC are found to be the kernel sizes, with declining importance from top to bottom layers, and special emphasis (IS > 0.6) for the first two layers: {K1, K2}. This can be visually tracked over the nonlinear trends $K_{i}=f\left(BAC\right)$ in Figure 6, where the median values of K1 and K2 exhibit the most substantial drop over the full BAC scale (e.g., *N* ≥ 3). Another comprehensive example illustrating K1 importance is the BAC colormap (Figure 5a), where K1 mid-range is the most definitive to maximal BAC. We turn the attention that kernel sizes of deep layers lose importance when the network reaches maximal shrink due to valid padding. This effect is observed for our deepest network (*N* = 7), where only limited search grid of K2–K7 ≤ 15 has been eligible, and therefore, its importance to BAC is practically reduced (IS < 0.3). In this case, the number of filters (F2–F5, *N* = 7) gains proportional importance (IS > 0.65). Generally speaking, in shallow networks (*N* < 7) the number of filters is the least important HPs except for the first layer F1 (IS = 0.3–0.35). This slight importance is confirmed in Figure 6 with the lack of visible trend in the distribution $F_{i}=f\left(BAC\right)$. Both Ki and Fi are related to the number of trainable parameters (Equation (2)), which is found to be the third most important feature to BAC (IS > 0.55, ranked after K1, K2). As shown in Figure 7, there is a decreasing trend in Params=f(BAC), which tends to an optimal minimal range of about 5000–20,000 parameters (also visible in the left top corner of the scatterplot in Figure 4a). We suggest that the importance scores highlighted in this study (Table 2) might reveal the CNN behavior in extracting abstract ECG features pertinent for the specific Sh/NSh classification setting, however, it is probable that the highlighted HPs have a generally high importance to the ability for model training and feature extraction in other ECG diagnostic applications.

Finally, the HPs of our optimal models with one to seven convolutional layers 

$HPopt={\left\{N,F_{i},K_{i}\;Param,LR\right\}}_{i=1}^{7}$ are presented in Table 3, subjected also to a learning rate optimization *LR* = [10^−5^; 10^−2^]. Generally, the optimal models with more than three convolutional layers do not exhibit substantial differences in performance BAC = (99.31%–99.5%). Among them, the best model HPbest is found with five convolutional layers, filters F={20,15,15,10,5}, kernel size K={10,10,10,10,10}, trained with default *LR* = 0.001, as specified in (Equation (8)). The best model has the smallest number of trainable parameters (7521) compared to others optimal candidates (with up to 187410 parameters) that is considered beneficial for the better generalization of the self-extracted abstract features with this model.

### 5.2. Analysis of Our Best CNN Model

The training and evaluation of our best model (HPbest) provide two general advantages:

- The use of two ECG sources from the most famous public ventricular arrhythmia databases and private OHCA databases provides а robust setting for model optimization on a large scale of ECG rhythms that can be seen by Holters and defibrillators during treatment of cardiac arrest patients. This article uses the largest number of (Sh + NSh) samples for training (720 + 3170) and validation (739 + 5921), which is the important precondition for design of robust deep learning shock advisory systems;
- The application of the model on ECG signals with short and long durations (2–10 s), with maximal performance for 5 s analysis (BAC = 99.5%, Se = 99.6%, Sp = 99.4%, Table 4) and tolerable drop in performance (<2% points) for very short 2 s analysis (BAC = 98.2%, Se = 97.6%, Sp = 98.7%, Table 4) can satisfy the crucial AED requirements for providing shock advisory decision with minimal hands-off delay after end of chest compressions.

### 5.3. Comparative Study to Other Published CNN Models for ECG Classification

The general purpose of this section is to present comparative study of our best model to a reference AED shock advisory system and other published fully-convolutional DNNs, originally recognized as state-of-the-art high-performance solutions to various ECG arrhythmia classification problems. Five CNN models were selected from literature with available information about their architectures and HPs configurations (Table 5). Two CNNs [53,60] had been originally designed for the same Sh/NSh detection purpose, one of them [60] presenting an LSTM recurrent layer in addition to two convolutional layers. The other three fully CNNs had been published for different ECG applications, including pulseless rhythm detection [56] and heartbeat classification [44,48]. This comparative study demonstrates the possibility of applying transfer learning through relearning of models designed for different ECG diagnostic purposes. We, however, could not test the original concept for transfer learning through reusing pretrained CNNs on ECG data for different purposes, as pretrained versions of the models in Table 5 are not publicly accessible.

**Figure 11:**
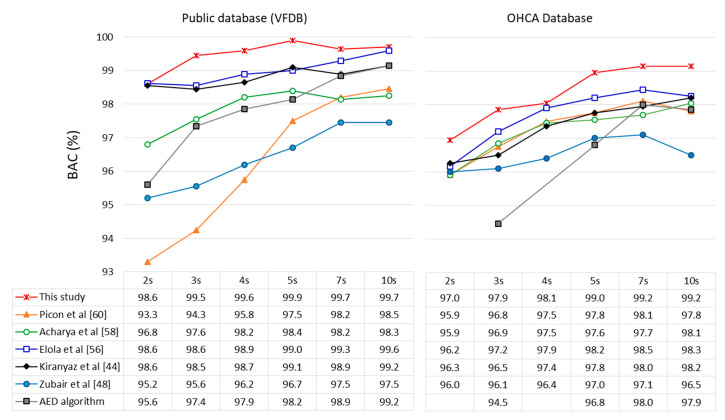
Comparative study of our best model to published fully convolutional DNNs, which are trained and evaluated under the same conditions on public Holter and OHCA databases. BAC performance is reported on our validation dataset using analysis durations between 2 s and 10 s. The performance of the reference automatic external defibrillator (AED) algorithm is reported for the same databases, taken from Krasteva et al. [19] for VFDB, Didon et al. [7] (3, 5, 7 s) and Krasteva et al. [25] (10 s) for the OHCA database.

A truthful comparative study was respected so that each CNN architecture in Table 5 was trained and evaluated under the same conditions, relying on the same data points, annotations, training and validation datasets from Holters and defibrillators, as used for training and validation of the best model in this study. All models were trained for input signals with durations 2–10 s and their BAC performance on the validation datasets is presented in Figure 11. The following conclusions can be drawn:

- Our best model outperforms all other models for both Public and OHCA databases. Its configuration can be distinguished as the deepest among others with five convolutional layers, while the number of filters and kernel sizes looks balanced within the middle range found in other studies. This result is a certain proof that HP optimization has an important role in accuracy and should always be carefully performed during DNN design for specific applications;
- The models of Elola et al. [56] and Kiranyaz et al. [44] are the next best models with up to about −1% points and −1.5% points’ drop in BAC, respectively. These models are good examples for successful transfer learning of CNNs in ECG signal processing, where CNN designs optimized for detection of pulseless rhythm and heartbeat classification are here successfully relearned for detection of shockable rhythms. The model of Elola et al. [56] can be distinguished as a deep model (four convolutional layers) with a small number of trainable parameters (1441, owing to the small number of filters and kernels), while the model of Kiranyaz et al. [44] can be distinguished as the shallowest model (two convolutional layers), but with the largest number of trainable parameters (8389 resulting from the largest number of filters and kernel sizes). These models are good examples to show that both deep and shallow networks can almost perform equally if their HPs are optimized in a specific ECG diagnostic application;
- The models of Picon et al. [60], Zubair et al. [48] and Acharya et al. [53] present the largest BAC drop (from −1% points to −5% points). The common HP setting observed in these models is the very small kernel size (three to five), which has been proven in our optimization study to have the most important impact to BAC (see Table 2);
- We note that the additional LSTM layer in the model of Picon et al. [60] provides evidence for inferiority, observing the considerable BAC drop (−2.5% points to −5% points) for short-duration signals < 5 s in Holter databases. This demonstrates that fully convolutional networks are indeed enough powerful to extract features for superior Sh/NSh detection performance at minimal computational cost than other DNN architectures.
- Our best model outperforms a reference shock-advisory system of a commercial AED (Fred Easy, Schiller Médical, France) based on hand-crafted ECG morphology features and a decision tree classifier [7,19,25] by about (+0.5% points to +3% points) for analysis durations of 10 s and 2 s, respectively. Indeed, the AED shock advisory system does not show inferior performance to three DNNs [48,58,60], which is a clear indication that unoptimized deep networks have no benefit compared to traditional machine learning algorithms.

## 6. Conclusions

This study presents the optimal HPs of deep CNNs with two to seven convolutional layers, derived by statistical justification of the common HPs of the top-ranked models. We observe a certain limit in the depth and width of CNNs design so that inferior performance is common for all shallow models (less than three convolutional layers), as well as deep (three to six layers) but very wide or very narrow models (highlighted importance for the kernel size of the first two layers). The deepest models (*N* = 7) might lose performance due to maximal model shrink, where the kernel size is fixed to minimum and the number of filters in deep convolutional layers become strong predictors of the CNN performance. The presented optimal HPs have a generally high importance to the ability for good model training and feature extraction, and might be efficient in other ECG diagnostic applications.

The general conclusion after evaluation in Holter and OHCA databases would be that our best CNN model provides very high Se (>95.2%) and Sp (>98.6%), even for very short analysis durations (2 s), and could be considered compliant with the AHA performance goals [4]. While the optimized network, based on fully convolutional hidden layers with pretrained weights can be run alone in real time without the need of preprocessing, waveform measurements, transformations, or other machine learning algorithms, it has perspectives for certain applications in unsupervised database annotation and diagnosis platforms, as well as in reliable real-time AED shock-advisory systems in OHCA.

## 7. Limitations

The size of the training/validation datasets is not divided according to the common scheme of 80%/20% as it isn’t a manually controllable factor but depends only on the number of cases found in independent data sources, predefined before the study. Thus, the cases are distributed uncommonly with majority of data for validation. The scheme with independent data sources is, however, respected to avoid the common cross-validation scenario without a patientwise control, where rhythms from the same patient are randomly shuffled in the training and validation datasets, and thus an overtraining on the specific patient rhythm is highly probable in deep learners.

From the methodological point of view, the HP optimization is done on the validation set, which is also used for evaluation. However, as soon as the study sets out to investigate the HP optimization itself, it is not an issue for the main conclusions, regarding the importance of HPs and comparison to other published DNN models, all trained and evaluated under the same conditions and datasets.

Although the presented study supports the derivation of valuable conclusions about the optimal depth and width of CNN models for Sh/NSh rhythm discrimination, we should note the inevitable influence of the size and content of the training and validation databases. Considering, however, the application of both public Holter and OHCA electrocardiograms, which cover the wide variety of Sh and NSh rhythms appearing during cardiac arrest and other critical situations and present as much as 3890 cases for training and 6660 cases for validation, we do not expect substantial differences in the context of Sh/NSh rhythm detection.

## Figures and Tables

**Table 1:** Number of 10 s strips in the training and validation datasets, collected from Holter and OHCA databases with respective arrhythmia annotations.

	Training Dataset				Validation Dataset		
Rhythm	AHADB	CUDB	OHCA1	Total	VFDB	OHCA2	Total
**VF**	430	93	66	**589**	308	221	**529**
**VT**	20	93	18	**131**	202	8	**210**
**NSR**	499	304	42	**845**	1023	154	**1177**
**ONR**	550	776	334	**1660**	1425	1063	**2488**
**ASYS**	6	4	655	**665**	4	2252	**2256**
**All Sh**	450	186	84	**720**	510	229	**739**
**All NSh**	1055	1084	1031	**3170**	2452	3469	**5921**

**Table 2:** Relative importance of $HPs={\left\{F_{i},K_{i},\;Params_{i}\right\}}_{i=1}^{7}\;$ as individual predictors of CNN performance. The importance range (0–1) is coded with a color gradient, highlighting the most important features (dense red).

N	F1	F2	F3	F4	F5	F6	F7	K1	K2	K3	K4	K5	K6	K7	Param
**1**	0.15							1.00							0.56
**2**	0.36	0.35						0.80	1.00						1.00
**3**	0.28	0.29	0.05					1.00	0.64	0.44					0.58
**4**	0.32	0.20	0.21	0.16				1.00	0.62	0.39	0.33				0.56
**5**	0.37	0.27	0.17	0.18	0.23			1.00	0.73	0.30	0.06	0.19			0.61
**6**	0.27	0.22	0.18	0.17	0.17	0.24		1.00	0.72	0.40	0.18	0.46	0.55		0.63
**7**	0.18	0.68	0.75	0.68	0.41	0.18	0.09	0.73	0.27	0.23	0.00	0.00	0.00	0.00	1.00

**Table 3:** Settings of the optimal HPs for CNNs with different numbers of convolutional layers: $HPopt={\left\{N,\;F_{i},K_{i}\right\}}_{i=1}^{7}$ The value of maxBAC is corresponding to maximal performance achieved after learning rate (LR) optimization (Figure 8). Note *: The highlight shows our choice for the best model (*N* = 5).

N	F1	F2	F3	F4	F5	F6	F7	K1	K2	K3	K4	K5	K6	K7	Param	LR	Max BAC
**1**	113							50							5877	0.001	96.70%
**2**	10	15						60	25						4391	0.001	98.99%
**3**	5	10	20					10	30	50					11606	0.001	99.31%
**4**	15	15	15	10				20	25	30	40				18741	0.001	99.31%
5 *	**20**	**15**	**15**	**10**	**5**			**10**	**10**	**10**	**10**	**10**			**7521**	**0.001**	**99.50%**
**6**	30	30	15	15	10	5		15	10	10	10	10	10		18311	0.0005	99.38%
**7**	40	30	25	15	10	5	5	15	5	5	5	5	5	5	13486	0.0005	99.45%

**Table 4:** Performance of our best CNN model with 2–10 s analysis durations, reported for all rhythms in different validation databases.

Se/Sp (rhythm)	Analysis Duration					
	2 s	3 s	4 s	5 s	7 s	10 s
Validation dataset: Total						
Se (all Sh), %	97.6	98.7	98.9	99.6	99.5	99.3
Sp (all NSh), %	98.7	99.1	99.3	99.4	99.5	99.7
**BAC, %**	**98.2**	**98.9**	**99.1**	**99.5**	**99.5**	**99.5**
Validation dataset: Holter (VFDB)						
Se (all Sh), %	98.6	99.4	99.8	100	99.8	100
Sp (all NSh), %	98.6	99.5	99.4	99.8	99.5	99.4
**BAC, %**	**98.6**	**99.5**	**99.6**	**99.9**	**99.7**	**99.7**
Validation dataset: OHCA2						
Se (all Sh), %	95.2	96.9	96.9	98.7	98.7	97.8
Sp (all NSh), %	98.7	98.8	99.2	99.2	99.6	99.1
**BAC, %**	**97.0**	**97.9**	**98.1**	**99.0**	**99.2**	**99.2**

**Table 5:** Configurations of published CNN networks for ECG arrhythmia classification, which are further subjected to training and evaluation with our databases for shockable or nonshockable rhythm (Sh/NSh) detection.

		CNN Layers				LSTM Layer	Dense Layers	
Methods	Original Application	N	Filters	Kernel Size	Max-Pool	Kernel Size	N (Kernel Size)	Trainable Params
This study	Sh/NSh detection	5	20, 15, 15, 10, 5	10, 10, 10, 10, 10	2	-	1 (2)	7521
Picon et al. [60]	Sh/NSh detection	2	32, 32	3, 3	7	20	1 (2)	7493
Acharya et al. [53]	Sh/NSh detection	4	3, 5, 10, 10	5, 5, 5, 4	2	-	3 (10, 5, 2)	939
Elola et al. [56] ^1^	Pulseless rhythm detection	4	8, 8, 8, 8	7, 7, 7, 7	2	-	1 (2)	1441
Kiranyaz et al. [44] ^1,2^	Heartbeat classification	2	32, 16	15, 15	6	-	2 (10, 2)	8389
Zubair et al. [48] ^1,2^	Heartbeat classification	3	32, 16, 8	5, 5, 5	2	-	1 (2)	3425

^1^ Transfer learning of CNN models used for other ECG classification tasks different than Sh/NSh detection. ^2^ The kernel size of the last dense layer was modified compared to the original publication to adapt for detection of two classes in our study.
